# Augmented reality-assisted microvascular decompression for glossopharyngeal neuralgia: a case report

**DOI:** 10.1093/jscr/rjae170

**Published:** 2024-03-22

**Authors:** Joshua Olexa, Annie Trang, Alhusain Nagm, Mohamed Labib

**Affiliations:** Department of Neurosurgery, University of Maryland School of Medicine, Baltimore, 10 MD, United States; Department of Neurosurgery, University of Maryland School of Medicine, Baltimore, 10 MD, United States; Department of Neurosurgery, University of Maryland School of Medicine, Baltimore, 10 MD, United States; Department of Neurosurgery, University of Maryland School of Medicine, Baltimore, 10 MD, United States

**Keywords:** augmented reality, glossopharyngeal neuralgia, microvascular decompression

## Abstract

Glossopharyngeal neuralgia is a rare condition characterized by pain along the distribution of the glossopharyngeal nerve. Surgical approaches via microvascular decompression represent a common treatment strategy. For this procedure, an understanding of the location of the cranial nerve and the offending vasculature is critically important. A mixed reality system was used to register patient-specific 3D models onto the patients head for operative planning and anatomical visualization. A 58-year-old female presented to neurosurgery with severe right-sided facial, tongue, and jaw pain unresponsive to multiple conservative therapies including medication. T2-weighted MRI with FIESTA sequence demonstrated right posterior inferior communicating artery compression of the right glossopharyngeal nerve entry zone. An augmented reality system was used to visualize the patients’ anatomy overlaid onto the patients’ head. A microvascular decompression of Cranial Nerves IX and X was performed via a retrosigmoid approach. Patient obtained significant relief of preoperative pain symptoms without complications.

## Introduction

A detailed anatomic understanding of the spatial relationships between offending vessels and cranial nerves is critical for surgical management of glossopharyngeal neuralgia [1, 2]. In the case of glossopharyngeal neuralgia with neurovascular compression, the most common offending artery is the posterior inferior communicating artery (PICA) [3]. Microvascular decompression (MVD) is the most common surgical treatment to create separation of the nerve and offending artery [4, 5].

Surgeons rely heavily on neuroimaging to confirm structural pathology of the patient’s clinical symptoms. New imaging technology and methods of visualization have continued to improve the surgeon’s understanding and operative approach to complex cranial cases. One such technology, augmented reality, has emerged recently as a novel method for interacting with and visualizing complicated neuroanatomy in three dimensions. Augmented reality superimposes digital content, such as 3D anatomical models onto the surgeon’s real-world view. The technology allows surgeons to visualize and manipulate 3D models from different perspectives as well as overlay the 3D model with the physical patient [6–11].

Herein, we describe the use of a novel markerless AR registration technology for operative planning of MVD for glossopharyngeal neuralgia. The AR system is an ultrafast, lightweight technology that precisely superimposes patient-specific 3D anatomy onto the head. This system provides a unique means of visualizing patient-anatomy, and the rapid registration process fits seamlessly into the clinical workflow. The system has the potential to improve localization and targeting of lesions and facilitates the development of a more comprehensive and accurate operative approach. Thus, this case report serves as a case study for the application of this technology in the setting of complex skull base anatomy in the surgical context of MVD for glossopharyngeal neuralgia.

## Case report

**Figure 1:**
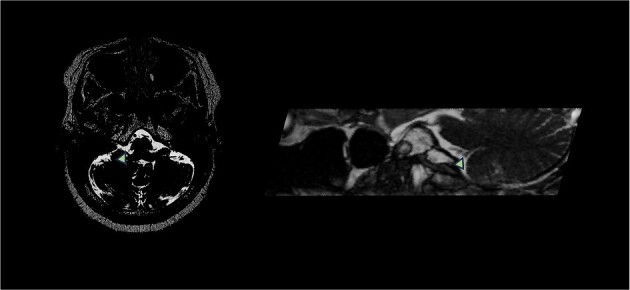
T2-weighted MRI with FIESTA sequence demonstrated right PICA compression of the right glossopharyngeal nerve entry zone demonstrating the axial and sagittal view. Arrow demonstrates location of neurovascular compression.

A 58-year-old female presented to neurosurgery with severe right-sided facial, tongue, and jaw pain unresponsive to multiple conservative therapies including medication. T2-weighted MRI with FIESTA sequence demonstrated right PICA compression of the right glossopharyngeal nerve entry zone (Fig. 1).

### Augmented reality technology platform

**Figure 2:**
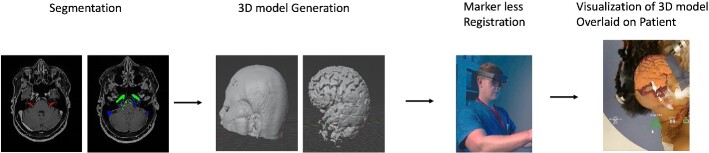
Overview of augmented reality system work flow. The process begins with segmentation of relevant anatomy followed by generation of a patient-specific 3D anatomical models. Using the headset, a surgeon registers the 3D model onto the patients’ head using a fiducial-less approach allowing for visualization of the 3D model overlaid onto the patients’ head.

The AR software application was developed by Hoth Intelligence (Philadelphia, Pennsylvania) and functions on the Microsoft Hololens 2 HMD (Redmond, Washington). The Microsoft Hololens 2 is an untethered optical see-through head-mounted display that superimposes virtual content (i.e. holograms, images, and screens) onto the users’ real-world field of view. A clinical workflow of the AR process is described in Fig. 2.

### Mixed reality registration

The AR technology used for this case overlays 3D digital models of the patient’s anatomy onto the head when viewed through the headset using a rapid, markerless (fiducial-less) registration process.

### Mixed reality presurgical planning

**Figure 3:**
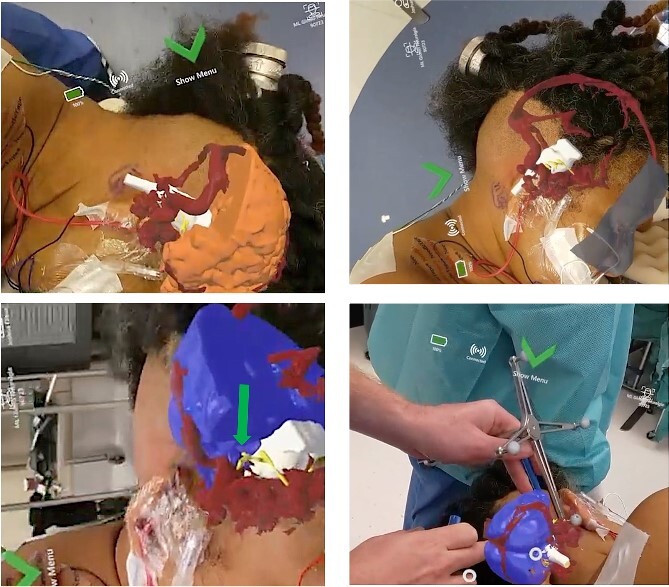
Representative views through the AR headset of 3D models overlaid onto the patient’s head from different perspectives. Brain (blue/orange), vasculature (red), cranial nerves (yellow), and brainstem (white).

After the patient was pinned in the operative position, the physician scanned the patient’s head while wearing the headset to overlay the patient’s 3D anatomical model onto their head (Fig. 3). The system was used in the operating room to visualize anatomy as well as assist with positioning and craniotomy planning. In particular, visualization of the glossopharyngeal nerve-PICA complex overlaid on the patient’s head. This allowed the user to better appreciate the physical interaction between PICA and the glossopharyngeal nerve and was used to inform incision planning.

### Operative and postoperative course

A retrosigmoid craniotomy for decompression of Cranial Nerves IX and X was performed. After opening the dura, microscopic visualization was used to open the cerebellopontine angle cistern and identify Cranial Nerves VII, IX, X, and XII. PICA was also identified. After failure to stimulate at 0.2 mA, Cranial Nerve IX was divided to visualize the root entry zone of Cranial Nerve X. Pledgets were placed between the PICA and the entry zones of Cranial Nerves IX and X before closure (Supplementary Video 1). Patient obtained significant relief of preoperative pain symptoms without complication and was discharged home with routine follow-up planned on postoperative Day 2.

## Discussion

In this report, we utilized the system in an MVD operation for treatment of glossopharyngeal neuralgia. We demonstrate the unique registration capabilities of the system and its value for preoperative planning. In this case, the surgeon was able to visualize the location of underlying anatomic structures—cranial nerves, brain tissue, vasculature, and brain stem—for incision and craniotomy planning.

Although this is not a technical report, it is worth noting unique advantages of the AR system used for this case. The system uses a markerless registration process. Fiducials are not required. The surgeon simply looks at the patient’s head, and computer vision algorithms place the 3D model in the proper location. Additionally, the registration time for this case was ~10 s. As such, the system fits seamlessly into the presurgical planning workflow without causing any delays. Lastly, and perhaps most critical, is the fact that the entire system operates solely out of the Microsoft Hololens 2 headset. Additional cameras, towers, or computers are not required. Altogether, the fast, markerless registration process and small footprint of the technology make it an ideal tool for surgical planning.

Several studies have described the use of augmented reality for complex cranial cases and have highlighted the benefits such as improved learning, decreased operative time, and favorable patient outcomes [6, 8, 12–14]. In general, the ease-of-use afforded by user-friendly visualization of 3D anatomy is well established. While there are notable technology differences between the various AR systems described in the literature, it is becoming increasingly apparent that AR can be a valuable surgical tool for complex neurosurgical cases.

This case report describes the first experience of incorporating AR into the presurgical planning of an MVD for glossopharyngeal neuralgia. While we describe the notable value of the system, we did not directly compare surgeries performed with and without AR. Future studies drawing these comparisons are warranted. Here we describe the use of an augmented reality system to aid in planning an MVD for glossopharyngeal neuralgia. The system provided the care team with a heightened understanding of the patient’s anatomy, and the ability to register the 3D models with the patient’s head was felt to be valuable in positioning, pre-surgical visualization, and incision planning.

## Supplementary Material

GN_VIdeo_rjae170 Display of 3D anatomical model overlaid onto the patients’ head as viewed through the AR headset.
